# Genetic variation and structure of endemic and endangered wild celery (*Kelussia odoratissima* Mozaff.) quantified using novel microsatellite markers developed by next-generation sequencing

**DOI:** 10.3389/fpls.2024.1301936

**Published:** 2024-04-04

**Authors:** Faezeh Mahdavikia, Mohammad-Taghi Ebadi, Abdolali Shojaeiyan, Mahdi Ayyari, Mohsen Falahati-Anbaran

**Affiliations:** ^1^ Department of Horticultural Science, Faculty of Agriculture, Tarbiat Modares University (TMU), Tehran, Iran; ^2^ NTNU University Museum, Norwegian University of Science and Technology (NTNU), Trondheim, Norway

**Keywords:** conservation genetics, endangered species, genetic diversity, *Kelussia odoratissima* Mozaff., microsatellite markers, natural habitats, next-generation sequencing (NGS), overexploitation

## Abstract

*Kelussia odoratissima* Mozaff. (Apiaceae) is a native plant that has been traditionally consumed in Iran’s food and pharmaceutical industries. Overharvesting of the taxon, especially at the beginning of the growing season, due to its considerable medicinal and economic value, is believed to be the main reason for the extirpating of this plant. The consequences of the severe anthropogenic impacts on the genetic diversity of populations are poorly known. In order to investigate the level of genetic variation and patterns of the genetic structure of *K. odoratissima*, we developed novel microsatellite markers using the 454 Roche next-generation sequencing (NGS) platform for the first time. Out of 1,165 microsatellite markers bioinformatically confirmed, twenty-five were tested, of which 23 were used to screen genetic variation across 12 natural populations. Our results showed that the average number of alleles per locus and the polymorphic information content (PIC) were 10.87 (range 7 to 27), and 0.81 (range 0.67 to 0.94), respectively. The mean observed and expected heterozygosities (± SD) across all populations were 0.80 ± 0.31 and 0.72 ± 0.14, respectively. The average pairwise *F_ST_* among the populations was 0.37 (range 0.04 to 0.81). Bayesian and distance-based clustering, and principal coordinate analyses revealed at least four major genetic clusters. Although high level of structure can be explained by landscape topography and geographic distance, presence of admixed populations can be associated to seed or pollen dispersal. Contrary to expectations, the high level of genetic variation and lack of inbreeding suggest that overexploitation has not yet significantly purged the allelic variability within the natural populations in protected areas.

## 1 Introduction

**Figure 1:**
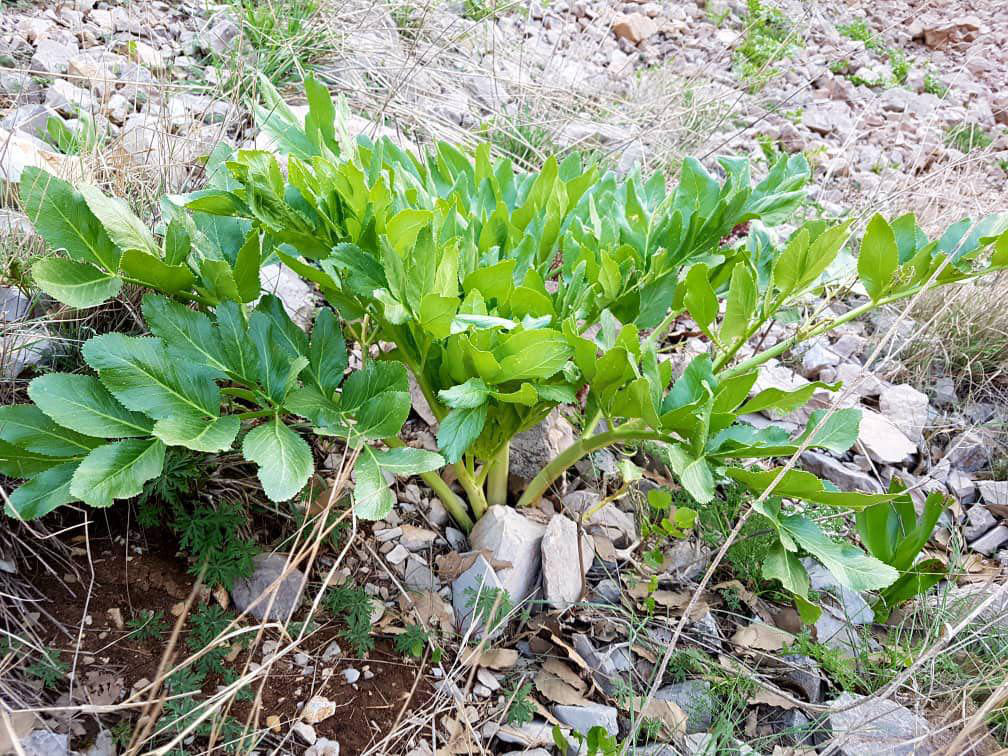
An individual of *Kelussia odoratissima* Mozaff. in the vegetative stage in the natural habitat. Photographed by F. Mahdavikia.

Mountain celery (*Kelussia odoratissima* Mozaff., **Figure 1**) already described as “*Amirkabiria odoratissima* Mozaff., *Apium graveolens* L., and *Opopanax* sp.” is the only species reported for *Kelussia* genus, the most recent genus introduced to Apiaceae (Mozaffarian, 2003, 2007). The plant is perennial herb and tall which is predominately outcrossing, pollinated mainly by bees (Jahantab et al., 2015). It inhabits alpine regions (found in over 2,500 m. a.s.l.) of central Zagros mountains (Iran) distributed in a restricted geographic range, experiencing subfreezing temperature for several months and thus short growing season (Mozaffarian, 2003). *Kelussia odoratissima* called locally as “Klous”, is consumed as a medicinal wild plant with a pleasant taste and aroma that contains valuable secondary metabolites including *Z*-Ligustilide and *Z*-Butylidene phthalide (Ahmadi et al., 2007; Ebrahimi et al., 2018). In addition, it contains total phenolic, flavonoid, and flavonol compounds, which are attributed to the cancer prevention and liver protection, and antibacterial and antimicrobial activity (Torki et al., 2018). Other pharmacological properties of this plant include analgesic, anti-diabetic, anti-inflammatory, anti-allergic, fibrinolytic, acid and pepsin reducing, and memory enhancing effects. In traditional medicine, the plant is used to treat disorders such as rheumatism, heart diseases, antispasmodics, menstrual pain relief, blood pressure, and cholesterol (Sajjadi et al., 2013; Hadian et al., 2014; Mirhosseini et al., 2015; Azizi et al., 2017; Ebrahimi et al., 2018; Torki et al., 2018).

The Zagros mountains range, is an important part of Iran’s endemic flora with high numbers of rare species, and is suggested as one of the areas with a high priority for preserving biodiversity (Omidbaigi et al., 2008; Noroozi et al., 2019). Our field observations of the natural habitats of *K. odoratissima* revealed that the plant has disappeared from several localities over the past two decades compared to what has been reported in the Flora of Iran (Mozaffarian, 2007). Our preliminary investigations suggest that this local extinction of the plant from natural habitats can readily be associated to direct and indirect anthropogenic activities. Although the plants in the protected areas can experience a full growing season and are able to reproduce normally, the plant is heavily harvested from the unprotected habitats due to the significant economic interest and their diverse range of culinary, medicinal, and other uses (Ramezani et al., 2022). Global climatic changes, inappropriate overgrazing, applying detrimental harvesting methods by local people might inevitably result in a reduction in population size or local extinction and consequently loss of genetic diversity (Ahmadi et al., 2019; Amiri et al., 2021). Although, the United Nations Development Program (UNDP) has considered this plant as an endangered species (Ahmadi et al., 2007), a comprehensive study is required to obtain information on the levels and patterns of genetic variation and structure of populations of *K. odoratissima*.

Developing and strengthening conservation genetic studies to assess or protect genetic diversity has been wildly suggested as a good strategy to assure the sustainable use of wild resources, especially endangered plants (Liu et al., 2020; Garcia-Jacas et al., 2021; Accogli et al., 2023; Perrino et al., 2023). The precise evaluations of genetic diversity and population genetic structure could assist to compile knowledge-based and efficient protection programs (Liu et al., 2020). Likewise, the absence of information on species’ genetic variation has frequently led the measures of conservation and restoration to defeat, and thus identifying populations with high genetic diversity and genetically dissimilar populations will be informative in the conservation programs (Garcia-Jacas et al., 2021). Loss of genetic diversity within populations is expected to decline their response to selection and increase the extinction probability of species (Aboukhalid et al., 2017). Therefore, adequate genetic variability is critical for minimizing inbreeding depression and thus boosting the adaptive potential of populations to overcome the environmental changes and assuring the persistence of species (Aboukhalid et al., 2017). Hence, assessment of the genetic diversity of isolated and rare species is considerably important when applying an appropriate conservation strategy to choose suitable populations and management approaches (Aboukhalid et al., 2017; Movahedi et al., 2019). Further, such genetic data can be linked to the ecological traits and also reconstructing the evolutionary history of natural populations (Aboukhalid et al., 2017; Movahedi et al., 2019).

Recently, the conservation of indigenous and threatened species has attracted considerable attention globally (Blyth et al., 2020; Coelho et al., 2020; Nzweundji et al., 2020; Perrino et al., 2022; Turco et al., 2023; Zhou et al., 2023). Investigations on the genetic diversity of vulnerable species by employing molecular markers have expanded in recent years because of their pivotal significance in planning for both *in-situ* and ex-situ conservation measures (Aboukhalid et al., 2017; Movahedi et al., 2019; Amiri et al., 2021). It is worth noting that DNA markers including single nucleotide polymorphisms (SNPs) and simple sequence repeats (SSRs or microsatellites) are powerful tools fundamental to the evaluation of diverse genetic parameters of plant populations (Palumbo et al., 2021). To develop SSR and SNP markers in non-model organisms, next-generation sequencing (NGS) techniques have broadly been utilized, because of being cost effective and less time consuming (Liu et al., 2020). Recently, conserved microRNAs of *K. odoratissima* have been identified using NGS data (Ramezani et al., 2022). Among different technologies, 454 Roche sequencing has wildly been utilized for SSR identification and development in several species including *Jatropha curcas* L., *Hevea brasiliensis* (Willd. ex A. Juss.) Müll. Arg., *Bituminaria bituminosa* (L.) C.H. Stirt (Pazos-Navarro et al., 2011; Salgado et al., 2014; Tian et al., 2017).

Simple sequence repeats consisting of 2 to 6 base pairs (bp) repetitive motifs are widespread across the genomes of all prokaryotes and eukaryotes. Microsatellite markers are utilized in a wide range of biological studies because of the high level of polymorphism, high abundance, co-dominance, high mutation rate, multi-allelic nature, high reliability at a relatively low cost, high reproducibility, and transferability across closely related species (Zhou et al., 2016; Liu et al., 2019, 2020; Wang, 2020; Garcia-Jacas et al., 2021). Most recently, a model and demonstration project called “Genetic Nature Reserves for Wild Celery (*Apium graveolens* L. and *Helosciadium repens* (Jacq.) W.D.J. Koch)” as a part of national network of genetic conservation areas in Germany has been launched to construct genetic reserves including 14 suitable wild populations of *Helosciadium repens*, a near-threatened wild celery species (Herden et al., 2020). The targeted populations displayed little genetic variation assessed by microsatellite markers (Herden et al., 2020). Moreover, in most studies investigating the level of genetic diversity and population structures, especially endemic and endangered plants, SSR markers have been marker of choice (e.g. *Seseli farrenyi* Molero & J. Pujadas (Garcia-Jacas et al., 2021), *Osmanthus serrulatus* Rehder (Chen et al., 2021), *Paeonia decomposita* Handel-Mazzetti (Wang, 2020), *Dalbergia odorifera* T. Chen (Liu et al., 2019), *Origanum compactum* L (Aboukhalid et al., 2017), and *Dendrobium officinale* Kimura & Migo (Xu et al., 2017). To our knowledge, despite all the advantages of SSR markers, no attempt has been undertaken to study the genetic diversity of *K. odoratissima*, and the only study conducted on the plant is limited to ISSR and sequence-related amplified polymorphism (SRAP) markers (Hadian et al., 2014).

The main aim of this study is to develop SSR markers in *Kelussia odoratissima* using the next-generation sequencing technology. These markers further are applied to determine the level of genetic diversity and population structure of this endangered indigenous taxon. The results might reveal the magnitude of allelic variation, and whether populations have experienced recent genetic bottleneck because of overharvesting.

## 2 Material and methods

### 2.1 Plant materials

*Kelussia odoratissima* Mozaff. mainly inhabits steep slopes of alpine regions in the central Zagros (Iran) where thin layers of soil exist. It is a perennial herb, found in alpine localities (1800 to 3163 m. a.s.l.) experiencing a cold semi-humid climate and grows in silty clay soil with high level of essential elements and organic matter (Jahantab et al., 2011). The emergence of the plant’s shoot occurs from early to late March, followed by flowering in July. Seed maturation takes place between August and the end of September (Mozaffarian, 2003; Ghasemi Nafchi, 2015).

Twelve populations were sampled from the entire distribution range of *K. odoratissima* in the Zagros mountains between May and June 2019 (**Table 1** and **Supplementary Figure S1**). As this species is endangered and severely harvested by local people, it is mainly found in nature reserves and protected areas. Therefore special permissions were acquired from the Iran Department of Natural Resources and Watershed Management Organization (No. 97/44/39291). Sampling without collecting any voucher specimens was performed with special precision and by collecting small amounts of leave tissue. Two fresh leaves from each individual were placed in a plastic bag containing silica gel. In total, 96 individuals were screened for allelic polymorphism using microsatellite markers.

**Table 1:** Geographic information of natural populations of *Kelussia odoratissima* Mozaff. used in this study.

Province	Locality	Abbreviation	No. Samples	Altitude (m)	Longitude (E)	Latitude (N)
Chaharmahal and Bakhtiari	SarAghaSeyed-I	Sras-I	8	1 853	49^°^49'33.6''	32^°^38'59.9''
Chaharmahal and Bakhtiari	SarAghaSeyed-II	Sras-II	8	1 896	49^°^48'02.4''	32^°^39'54.3''
Chaharmahal and Bakhtiari	SarAghaSeyed-III	Sras-III	8	1 827	49^°^47'27.8''	32^°^40'22.4''
Chaharmahal and Bakhtiari	Birahgan	Brgn	8	2 276	49^°^58'13.1''	32^°^40'48.6''
Isfahan	Abdoz	Abdz	8	2 711	49^°^53'32.0''	32^°^45'53.7''
Isfahan	Kelose	Klse	8	2 802	49^°^50'54.3''	32^°^46'57.7''
Isfahan	DareSepestan	Drsp	8	2 531	49^°^53'8.2''	32^°^47'12.9''
Isfahan	Vestegan	Vstg	8	2 779	49^°^55'19.5''	32^°^43'48.6''
Isfahan	Kahgan	Khgn	8	3 040	49^°^57'55.6''	32^°^43'60.0''
Isfahan	Durak	Durk	8	2 280	49^°^53'52.6''	32^°^40'54.5''
Kohgiluyeh and Boyer-Ahmad	DelAfruz	Dlfz	8	2 818	50^°^26'11.3''	31^°^22'22.0''
Khuzestan	Gharun	Ghrn	8	3 200	50^°^15'22.0''	31^°^27'39.3''

### 2.2 Sequencing, bioinformatic analysis, and amplification of microsatellite loci

The genomic DNA was extracted from dried leave tissue by employing the ENZA SP Plant DNA Kit (Omega Bio-Tek, Norcross, USA) following the manufacturer’s protocol. In order to determine DNA concentration and quality, spectrophotometric quantification method (Epoch, BioTek, USA) was used along with 2% agarose gel electrophoresis. Microsatellite libraries were generated by pooling genomic DNA of two individuals from all populations. Fragmented DNA were hybridized with a number of distinct probes containing motifs with different sizes (TC, TG, AAC, AGG, AAG, ACG, ACTC, and ACAT) for enrichment of microsatellite loci (Malausa et al., 2011). Sequencing of the enriched microsatellite library was conducted with 454-GS-FLX sequencer in accordance with the manufacturer’s guidelines (Roche Diagnostics, France). Roche 454 sequencing technique yielded 25,423 reads with length of at least 80 nucleotides. To identify sequences containing microsatellite motifs, alignment of reads and primer designing were performed with QDD version 3 with default settings (Meglécz et al., 2009). Out of 1,165 bioinformatically confirmed loci,195 primer pairs were selected as best candidates containing di-, tri-, tetranucleotide repeat motifs (GC content between 40-60% and melting temperature, Tm, between 60-70°C). Twenty-five primer pairs were selected to test for amplification in 8 samples from each of twelve populations. Twenty-three loci were successfully amplified with expected allele sizes visualized with 2% agarose gel electrophoresis. The sequence data of amplified loci were submitted to GenBank at the National Center for Biotechnology Information (accession numbers OQ992688- OQ992710, **Supplementary Table S1**).

PCR amplification was carried out in 7 μl reactions containing 3.5 μl of 2×Taq DNA Master Mix Red (Ampliqon, Denmark), 10 pmol of each forward and reverse primers, and 0.7 μl of genomic DNA in a C1000™ Thermocycler (Bio-Rad, USA). The thermal profile consisted of initial denaturation at 95°C for 5 min followed by 10 cycles as touchdown in 95°C for 30 s, 64-53°C for 30s (1°C decrease in each cycle), 72°C for 30 s, and 25 cycles in 95°C for 30 s, 53°C for 30s, 72°C for 30 s followed by a final extension at 72°C for 5 min (**Supplementary Table S1**).

To determine the size of the alleles at microsatellite loci, PCR products were electrophorized using a 10% denaturing polyacrylamide and were visualized by the silver staining methods (Bassam and Gresshoff, 2007; Huang et al., 2018). The size of alleles was determined by using DM1100 ExcelBand™ 50-bp DNA ladder (SMOBIO Technology).

### 2.3 Statistical analyses

Allelic polymorphism was screened using 23 amplified loci across natural populations of *K. odoratissima*. The polymorphic information content (PIC) for each locus was computed using allelic frequency throughout the entire individuals with CERVUS version 3.0.7 (Kalinowski et al., 2007). The effective number of alleles and the number of different alleles per locus was determined using GenAlEx version 6.501 (Peakall and Smouse, 2006). Private allelic richness and allelic richness for each population were computed with HP-rare version 1.0 (Kalinowski, 2005). The observed (H_O_) and expected (H_E_) heterozygosity and inbreeding coefficient (*F_I_*_S_) were estimated with Genepop version 4.7.5 (Rousset, 2008). The Hardy–Weinberg equilibrium for each population was tested, and the statistical significance was determined using a Markov chain method. A bottleneck test to explore the departure from mutation-drift equilibrium was performed with the stepwise mutation model (SMM), a two-phase mutation model (TPM) and mode-shift test using BOTTLENECK version 1.2.02 (Piry et al., 1999). The patterns of genetic differentiation among populations were determined with both model-based Bayesian clustering and distance-based clustering analyses. Pairwise *F_ST_* between populations was estimated using Genepop. To partition the total genetic variation into within and between populations, the analysis of molecular variance (AMOVA) was performed with Arlequin version 3.5.2.2 (Excoffier and Lischer, 2010).

A Neighbor-Joining tree was constructed based on Nei et al.’s Da distance using Population 1.2.32 software (Langella, 1999; Nei et al., 1983). A network-based graph was constructed with SplitsTree V5 to show the genetic relationship between individuals with the Neighbor-net algorithm (Bryant and Moulton, 2004). In addition, principal coordinate analysis (PCoA) was performed based on Da genetic distance using GenAlEx 6.51 (Peakall and Smouse, 2006). To determine the pattern of isolation by distance (IBD), correlation between geographic and genetic distances was examined with Mantel test using GenAlEx (Peakall and Smouse, 2006).

To determine the pattern of genetic structure and the level of admixture among populations of *K. odoratissima*, a model-based structure analysis was applied using STRUCTURE version 2.3.4 (Pritchard et al., 2000). To estimate genetic parameters, a 2 × 10^5^ burn-in period followed by 10^5^ Markov Chain Monte-Carlo (MCMC) iterations was used for a number of predefined number of clusters, K, ranging from 1 to 13, each with 16 independent runs. The most likely number of genetic clusters was ascertained by computing the ΔK (Evanno et al., 2005) and by depicting the mean ln estimated probability of data using Structure Harvester (Earl and vonHoldt, 2012). To illustrate the level of structure by visualizing the proportion of assignment of each individual to each genetic cluster, ClumpAK algorithm was utilized to summarize the results of most similar runs (Kopelman et al., 2015).

## 3 Results

### 3.1 Development of SSR markers

Analysis of microsatellite motifs revealed that dinucleotide repeats constituted 68.7% of all reads varying in the number of repeats ranging from 4 to 25 and the AC motif being the most frequent among all repeat types. In addition, trinucleotide and tetranucleotide repeats constituted 30.2% and 1.0% of total motifs, respectively (**Supplementary Table S2**, **Figure S2**).

Total number of alleles detected across 96 individuals were 250, with a mean of 10.87 alleles per locus (ranging from 7 to 27; **Supplementary Table S3**). All loci showed considerably high polymorphic information content (PIC > 0.6), ranging from 0.67 (K.odora06) to 0.94 (K.odora21) with a mean of 0.81 per locus (**Table 2**). The average observed and expected heterozygosity over all populations were 0.80 ± 0.31 (range 0-1) and 0.72 ± 0.14 (range 0-0.93), respectively. The range of allelic richness (Rs) and private allelic richness (Rp) were 3.51 to 4.06 (average = 3.7) and 0.08 to 0.56 (average = 0.17), respectively. A high Rs (4.06) and Rp (0.56) were observed in Sras-II and Ghrun populations, respectively (**Supplementary Table S3**, **Table 2**). Most of the study populations showed heterozygosity deficiency as a consequence of departure from mutation-drift equilibrium with both TPM and the SMM models (Wilcoxon rank test, *P*> 0.05) (**Table 2**).

**Table 2:** Average values of genetic statistics of 23 microsatellite loci with the population bottleneck probabilities for 12 populations of *K. odoratissima* under the two models (SMM and TPM).

Population	Na	H_O_	H_E_	*P*	*r*	N_E_	R_S_	R_P_	*F*_IS_	TPM	SMM	Mode-shift
Sras-I	4.348	0.783	0.738	0.134	0.077	3.563	3.7	0.08	-0.105	0**	0.00002**	shifted mode
Sras-II	5.174	0.811	0.758	0.121	0.057	3.876	4.05	0.14	-0.099	0.00615**	0.090	L-shaped
Sras-III	4.348	0.776	0.708	0.106	0.085	3.497	3.67	0.23	-0.126	0.00002**	0.00107**	shifted mode
Brgn	4.130	0.793	0.706	0.067	0.083	3.382	3.57	0.13	-0.147	0**	0.00002**	shifted mode
Abdz	4.652	0.787	0.740	0.114	0.070	3.712	3.87	0.14	-0.147	0.00064**	0.00558**	shifted mode
Klse	4.043	0.853	0.698	0.097	0.048	3.361	3.51	0.15	-0.264	0**	0**	shifted mode
Drsp	4.522	0.803	0.725	0.106	0.051	3.546	3.7	0.1	-0.125	0**	0.00042**	shifted mode
Vstg	4.696	0.814	0.710	0.102	0.052	3.555	3.75	0.1	-0.193	0.01166*	0.12337	L-shaped
Khgn	4.739	0.848	0.716	0.126	0.040	3.681	3.83	0.12	-0.24	0.00008**	0.00615**	L-shaped
Durk	4.217	0.766	0.694	0.127	0.048	3.240	3.61	0.1	-0.118	0.00025**	0.01794*	shifted mode
Dlfz	4.826	0.831	0.740	0.089	0.058	3.798	3.91	0.18	-0.181	0.00024**	0.01384*	shifted mode
Ghrn	4.696	0.789	0.704	0.068	0.063	3.607	3.74	0.56	-0.119	0.00002**	0.01043*	shifted mode

Na, H_O_, H_E_, r, P, Ne, Rs, Rp, *F_IS_* denote the number of alleles, observed and expected heterozygosities, frequency of null alleles, *P*-value for the HW equilibrium test, the effective number of alleles, allelic richness, private allelic richness, and inbreeding coefficient, respectively.

*: significant (*P* < 0.05), and **: significant (*P* < 0.01).

### 3.2 Inter-population genetic differentiation and genetic structure

**Figure 2:**
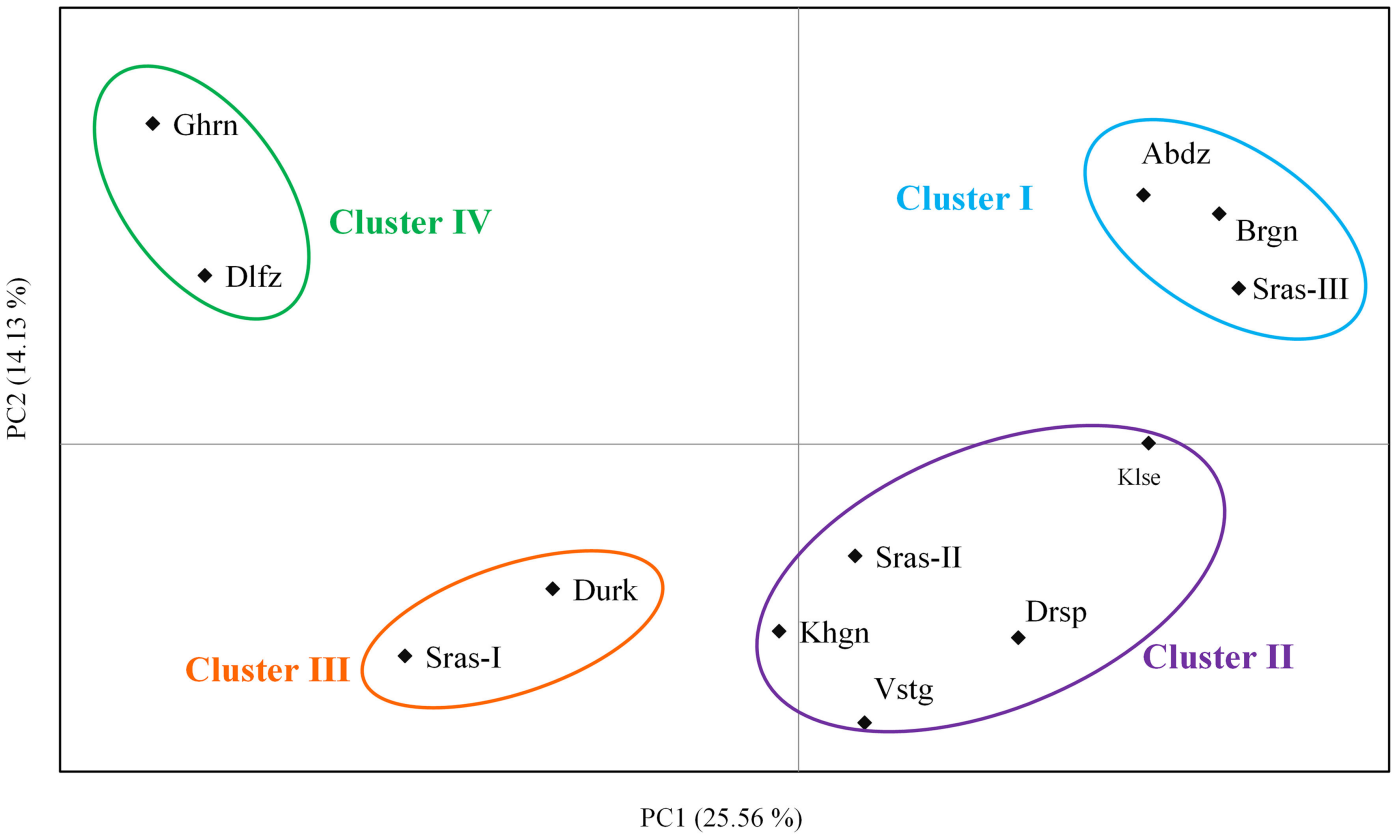
The two-dimensional plot obtained from principal coordinate analysis (PCoA) based on the first two coordinates in *K. odoratissima*. Populations are grouped based on 4 major genetic clusters supported by Bayesian structure analysis.

**Figure 3:**
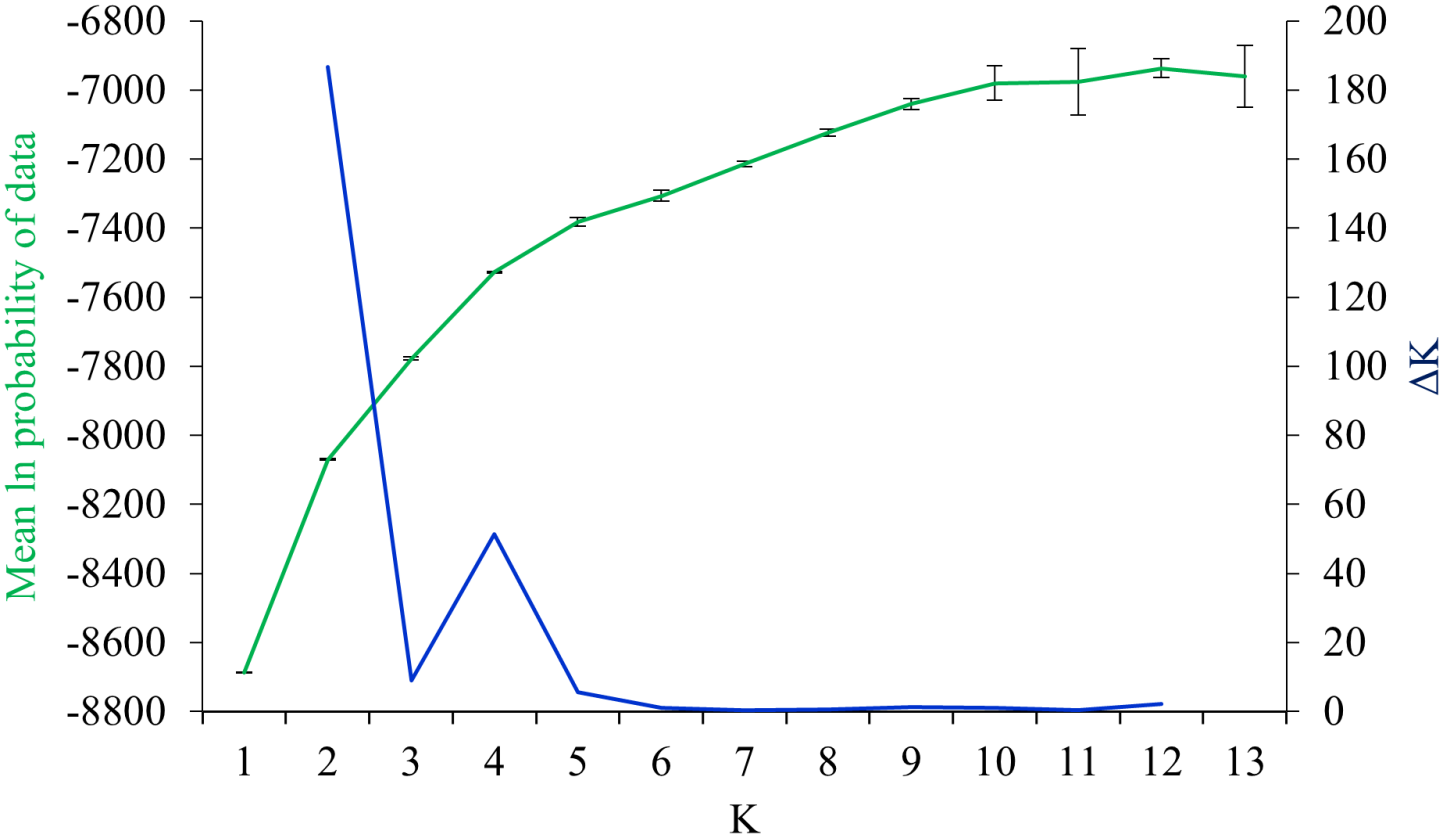
The plot of ΔK statistic and mean estimated ln probability of data ( ± SD) obtained from 16 independent runs for each K.

**Figure 4:**
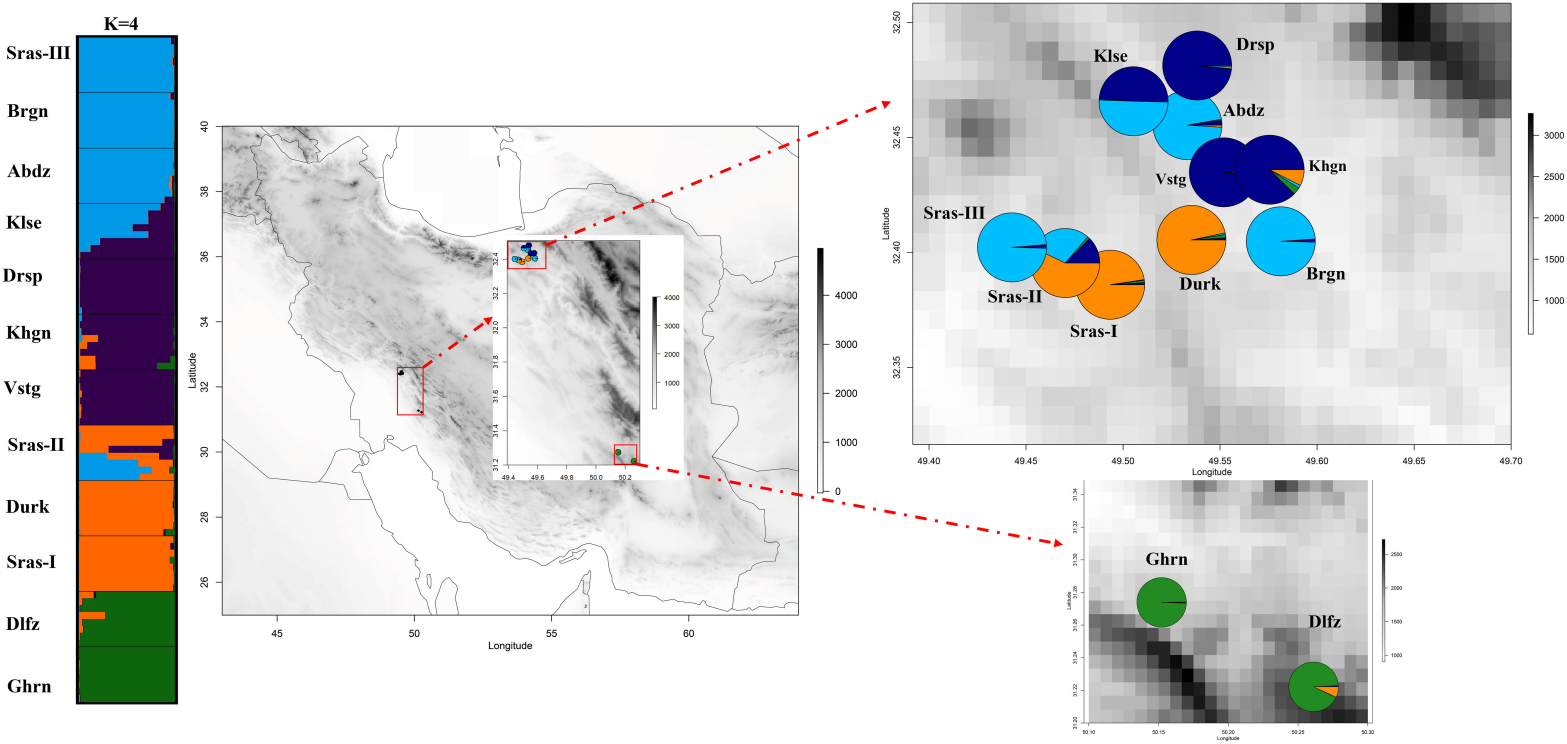
The genetic structure of 96 individuals from natural populations of *K. odoratissima*’s. The membership proportion of individuals (left) and populations (right) to each genetic cluster. The membership coefficient are obtained from summarizing the outputs of 16 separate runs with high similarity scores (K4=0.99).

**Figure 5:**
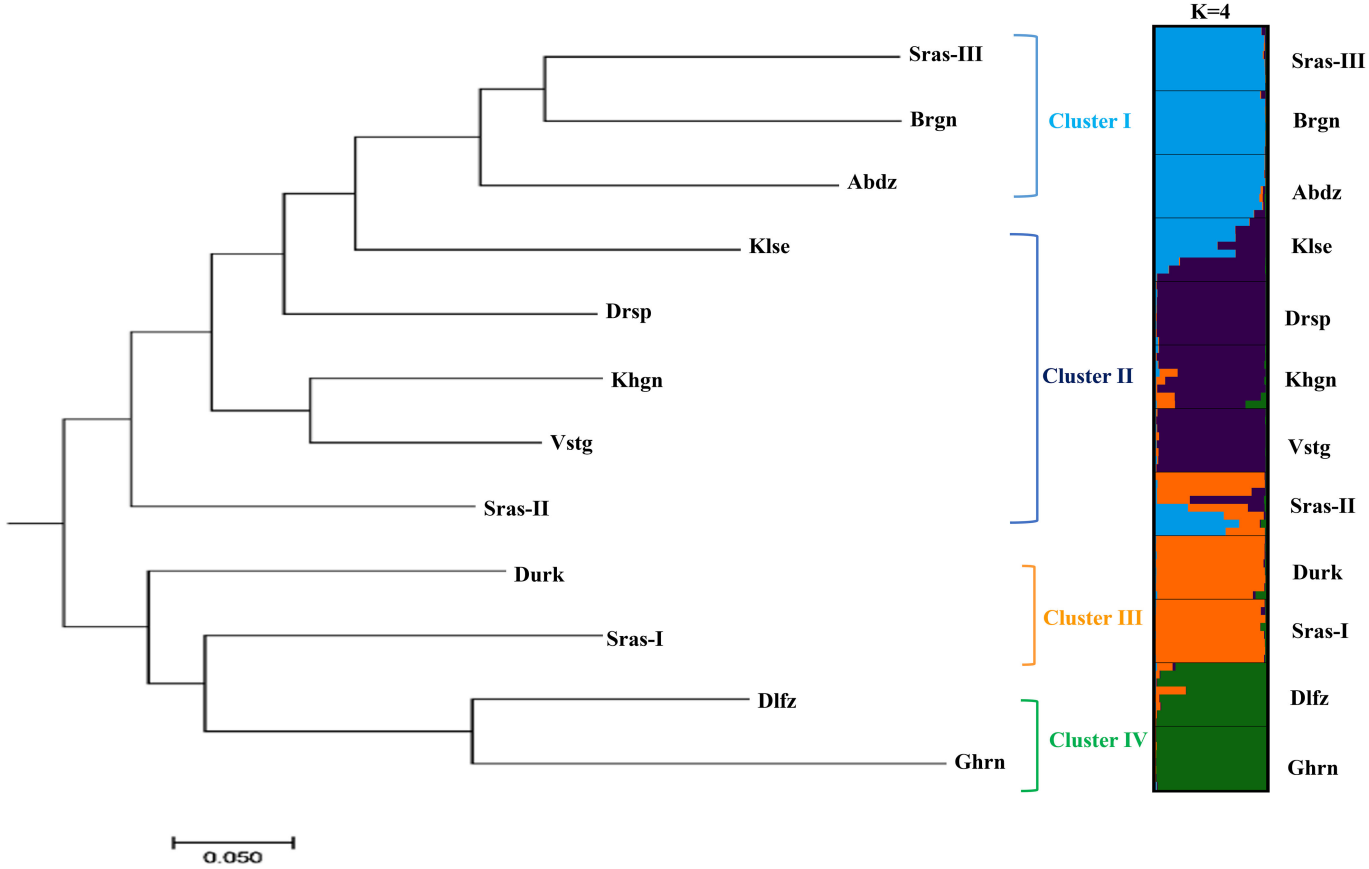
The neighbour-joining tree (left) obtained from was obtained Nei et al. (1983) Da genetic distance for 12 populations of *K. odoratissima.* The membership proportion of populations (right) to each genetic cluster (right).

**Figure 6:**
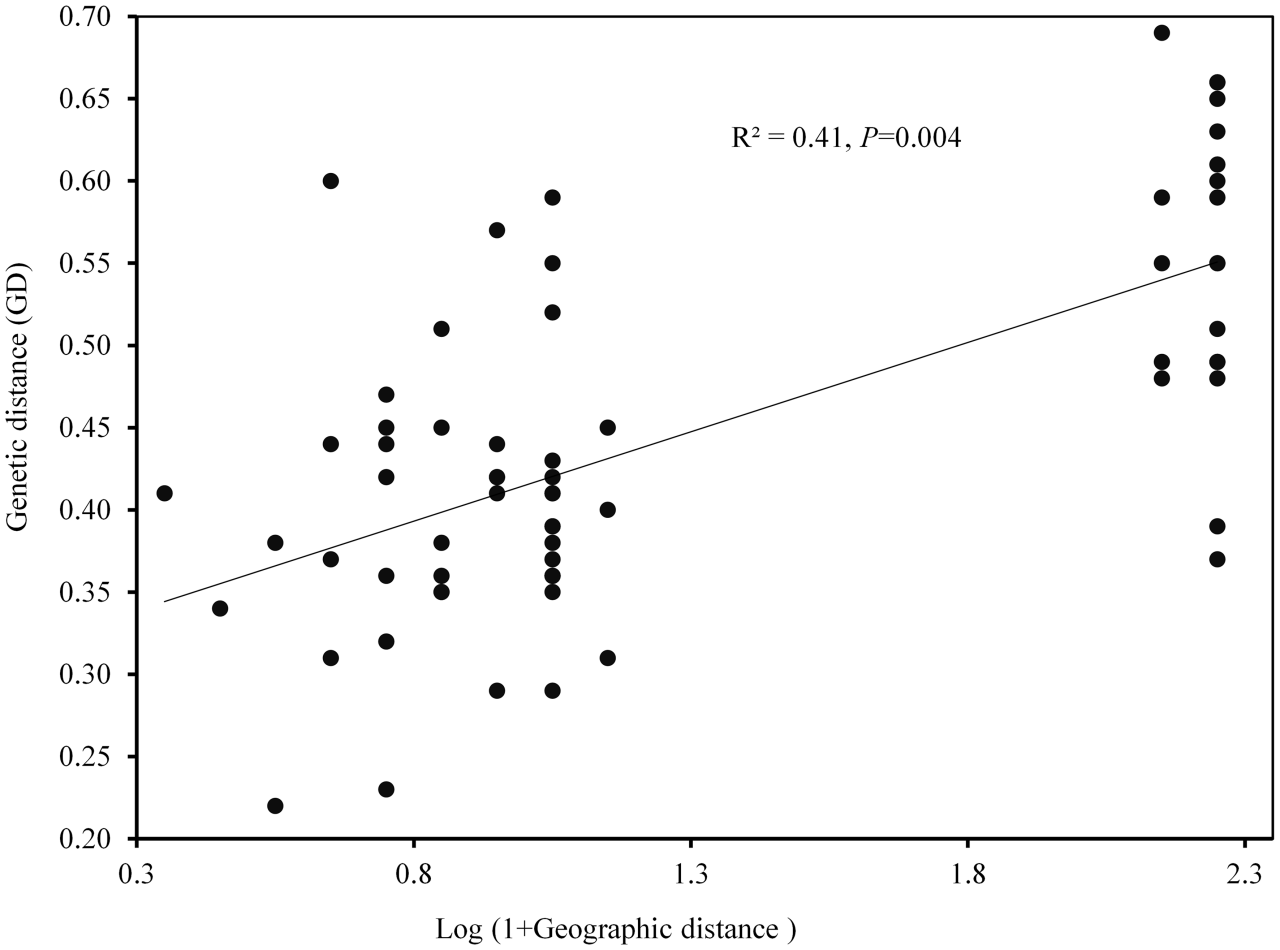
The relationship between geographic and genetic distance for natural populations of K. odoratissima (0.64, P < 0.05).

The AMOVA demonstrated that most genetic variation (88%) partitioned within populations, whereas only 12% of total variation is accounted for among populations differentiation (**Supplementary Table S4**). The pairwise *F_ST_* among populations of *K. odoratissima* ranged from 0.04 to 0.81 (average *F_ST_* =0.37). The results of PCoA revealed that the first two principal coordinates constituted 25.56% and 14.13% of the total variation (**Figure 2**), and four distinct genetic groups were identified.. The Bayesian clustering analysis based on ΔK and the mean ln estimated possibility of data exhibited the optimum number of genetic clusters at K=2 and K=4, respectively (**Figure 3**). Several populations of *K. odoratissima* including Sras-II, Sras-III, Brgn, Abdz, Klse, Drsp, Vstg, and Khgn (Clusters I and II) were diverged from the rest of populations (K=2, Clusters III and IV, **Supplementary Figure S3**). Three populations including Drsp, Vstg, and Khgn, divided in a separate cluster at K=3. Two populations Sras-II and Klse represented some degree of admixture originating from clusters I and III, respectively (**Figure 4**). Additionally, the result of genetic distance-based clustering analysis including neighbour-joining (NJ) tree revealed a similar pattern of structuring across natural populations of *K. odoratissima* (**Figure 5**). In the split graph created by the neighbor-net analysis, all individuals of the populations Sras-III, Brgn, and Abdz except Abdz-08 were clustered in a separate cluster (**Supplementary Figure S4**). The populations from Khgn and Sras-II (excluding Sras-II-01) were closely connected to the populations from Klse, Drsp, and Vstg, as well as one individual from the population Abdz (Abdz-08). Sras-I, Durk, Dlfz, and Ghrn populations were also grouped with one member of population Sras-II (Sras-II-01). The result of the Mantel test showed a significant relationship between geographic and genetic distances (r=0.64, *P* < 0.05, **Figure 6**).

## 4 Discussion

One of the most substantial elements of the research on conservation of scarce and endangered plant species is having knowledge on the level of genetic diversity which is important factor determining the adaptability of species to the environment (Ghafouri and Rahimmalek, 2018; Rameshkumar et al., 2019; Liu et al., 2019; Wu et al., 2020; Yang et al., 2022; Zhang et al., 2022; Rahali et al., 2016; Garcia-Jacas et al., 2021). Our study shows that microsatellite markers might be well suited for conservation genetics by determining genetic diversity and structure of endemic and endangered species because of high variability among individuals within populations, thus capable of identifying different genetic source of populations (Wu et al., 2020; Garcia-Jacas et al., 2021).

Analysis of microsatellite motifs showed that the most abundant types of di- and tri-nucleotide repeats are AC/GT, CA/TG, TTC/GAA, and GAA/TTC, and the number of repeats considerably varies between the various SSR repeat types. Comparing the studies carried out on the several species of the Apiaceae family including *Notopterygium incisum* C. C. Ting ex H. T. Chang, *Apium graveolens*, *Cuminum cyminum* L., *Anethum sowa* Roxb. Ex Fleming, and *Heracleum* sp. revealed that AT/AT, GA/TC, AAG/CTT, and TGA/TCA were the most predominant motifs among dinucleotide and trinucleotide motifs (Fu et al., 2013; Bharti et al., 2018; Jia et al., 2019; Daemi-Saeidabad et al., 2020; Kumar et al., 2020). These discrepancies could be explained by the difference in genomic content and structure of the investigated species, marker development techniques, and mining principles (Liu et al., 2019; Lopez et al., 2015; Zhang et al., 2017; Zhang et al., 2022).

Our results showed high variability in the number of alleles among microsatellite loci. The high PIC value (ranging from 0.67 to 0.94) suggests that the developed markers were highly polymorphic implying their superior discriminatory power and the diverse genotypes used in this research (Verma et al., 2019). In contrast, the number of microsatellite alleles per locus and PIC values in celery ranged from 2 to 4 (mean 2.68) and 0.06 to 0.72 (mean 0.35), respectively (Fu et al., 2013). The number and variety of SSR markers used, the number of individuals, the diversity of plant material, and the type of their dispersal, could explain these discrepancies (Bharti et al., 2018; Carvalho et al., 2019; Liu et al., 2019; Verma et al., 2019).

The level of expected heterozygosity (H_E_=0.72) quantified in *K. odoratissima* was higher than the reference value for other endemic species (0.42) (Wang, 2020; Garcia-Jacas et al., 2021). Similar studies in Apiaceae family have also reported a high level of heterozygosity (e.g. *Cuminum cyminum* (Bharti et al., 2018) and *Angelica dahurica* Bai Zhi (Liu et al., 2020). In contrast, a low level of observed heterozygosity (H_O_=0.14, range 0 – 0.92) and expected heterozygosity (mean=0.36, range 0.06 – 0.74) is estimated for *Apium graveolens* accessions (Fu et al., 2013). Similarly, a low level of observed and expected heterozygosities (H_O_=0.172 and H_E_ =0.303, respectively) has reported in *Foeniculum vulgare* Mill (Aiello et al., 2020). The high level of genetic variation observed in *K. odoratissima* is probably due to outcrossing mating system of the plant (F_IS_ values close to zero), mainly pollinated by bees (Jahantab et al., 2015) and thus limited number of selfed progeny. Other factors such as perenniality and persistent soil seed bank may explain this high level of genetic diversity in this species which make it less sensitive to the risk of the depletion of genetic diversity (Falahati-anbaran et al., 2011; Aboukhalid et al., 2017). A high genetic diversity is expected to weaken the level of inbreeding depression and this improves population’s ability to adapt to changes in the environment (Schäfer et al., 2020). Finally, the existence of genetic diversity in the populations of rare species has a significant advantage and should be considered in planning conservation programmes (Mcdonnell et al., 2021).

The result of the mutation-drift equilibrium test revealed heterozygosity excess all of the *K. odoratissima* natural populations, suggesting that populations might have recent experienced bottleneck events. Similar findings have reported presence of bottleneck in *Populus wulianensis* S.B.Liang & X.W.Li and *Osmanthus serrulatus* populations (Wu et al., 2020; Chen et al., 2021). In our study, few populations including Sras-II, Vstg, and Khgn populations exhibited L-shaped distributions of alleles, indicating that these areas may have not experienced drastic bottleneck. It is worth mentioning that one should not neglect the profound impacts of anthropogenic activity on this medicinal plant. Identifying recently bottlenecked populations is incredibly important because populations might have not still been capable to cope with population size decline, and consequently might be in danger of extinction (Luikart et al., 1998; Aboukhalid et al., 2017). Thus, the earlier a bottleneck is identified, the more likely it is to reduce the bottleneck’s devastating effects through the implementation of mitigation management processes such as habitat protection and restoration or the introduction of migrants (Luikart et al., 1998).

AMOVA analysis revealed that a small portion of genetic variation is attributed to among population differentiation. High level of molecular variation occurred within populations found in our study is somewhat in congruent with previous studies conducted based on diverse molecular markers on some species of the Apiaceae family and other endangered and endemic species in other parts of the world such as *Trachyspermum ammi* (L.) Sprague (Fadaei Heidari et al., 2016), *Ducrosia anethifolia* (DC.) Boiss (Sabbaghi Rahimi and Rahimmalek, 2019), *Ferula asafoetida* H. Karst (Tajbakht et al., 2018), *Osmanthus serrulatus* (Chen et al., 2021), *Populus wulianensis* (Wu et al., 2020), *Panax vietnamensis* Ha et Grushv (Vu et al., 2020), *Origanum compactum* (Aboukhalid et al., 2017), *Dalbergia odorifera* (Liu et al., 2019), and *Paeonia decomposita* (Wang, 2020). High within populations variation can be attributed to various mechanisms including outcrossing pollination system, seed and pollen dispersal and also high historical effective population size (Falahati-anbaran et al., 2011; Ghafouri and Rahimmalek, 2018; Sabbaghi Rahimi and Rahimmalek, 2019). A significant correlation between genetic and geographic distances found in our study, demonstrates strong isolation by distance shaping the genetic differentiation among *K. odoratissima* populations. This finding was congruent with the results reported by Wang (2020) for *Paeonia decomposita*, and Yang et al. (2022) for *Monochasma savatieri* Franch. Ex Maxim. In contrast, no isolation by distance have been reported for *Allium sativum* L (Li et al., 2022), *Fragaria nilgerrensis* Schlecht (Lu et al., 2021), and *Origanum compactum* (Aboukhalid et al., 2017). Taken together, our results suggest that gene flow among closely located populations as a predominant factor shaping genetic variation within and among populations.

### 4.1 Genetic structure of *K. odoratissima*

Both distance-based clustering and Bayesian clustering analyses supported 4 major genetic clusters in the study populations of *K. odoratissima.* The populations located in the southernmost distribution range were genetically differentiated from majority of the northernmost populations. In K=3, populations from the northern region were separated into two distinct groups, and in spite of limited gene flow among most populations, there was some evidence of admixture in Klse and Sras-II, each exhibiting genetic mixture of Clusters I and II and Clusters II and III, respectively. Almost both admixed populations arose from combination of genetically diverged populations that were only a few kilometres apart (Sras-II between Sras-I and Sras-III; Klse between Abdz and Drsp). Also, the southernmost populations, Dlfz and Ghrn, showed distinct genetic ancestry compared to the populations located in the northern region (**Figures 4**, **5**). Although, no research regarding pollination biology or outcrossing rate have been done in *K. odoratissima*, its flower structure and frequent pollinator visits, might suggest high levels of cross-pollination by insects, which is common in other species of Apiaceae (Aboukhalid et al., 2017; Palumbo et al., 2021). *Kelussia odoratissima*’s umbles usually consist of fertile hermaphrodite flowers and sterile lateral flowers (Mozaffarian, 2003). Pollinator differences in mobilities, behaviour and mechanics of pollen transfer create specific patterns of gene flow both within and between populations of the plants (Wessinger, 2021). For example, some natural barriers like the mountains’ topography and geographic structure may prevent pollinating insects from flying, which would reduce connectivity between populations (Tajbakht et al., 2018). It can be speculated that the admixture in the natural *K. odoratissima* populations, similar to other species of this family, could be attributed to seed/pollen dispersal which is facilitated by longer flowering period and pollination efficiency (Carvalho et al., 2019; Sabbaghi Rahimi and Rahimmalek, 2019). Similar admixture patterns have also been observed in *Trachyspermum ammi* accessions (Fadaei Heidari et al., 2016). High level of structure and admixture have also been in *Ducrosia anethifolia* (Apiaceae) and *Ferula asafoetida* populations (Tajbakht et al., 2018; Sabbaghi Rahimi and Rahimmalek, 2019). Taken together, our results are in consistent with other studies that have identified multiple genetic clusters in various endemic and threatened species like *Dalbergia odorifera*, *Torreya grandis* Fort. Et Lindl., *Satureja bachtiarica* Bunge, *Origanum compactum*, and *Paeonia decomposita* (Aboukhalid et al., 2017; Zeng et al., 2018; Liu et al., 2019; Movahedi et al., 2019; Wang, 2020).

As mentioned before, some ecological and environmental factors might act as a natural barrier against gene flow in some nearby populations (Kazemeini et al., 2020). Several different factors including genetic drift, limited gene flow, and local adaptation could cause diversification in endangered endemic species. Thus, additional research is required to assess the significance of different factors impacting genetic variation in order to conserve the natural populations of *K. odoratissima.* Our results demonstrate that landscape topography and geographic distance could explain some of the genetic structure found in the natural population of *K. odoratissima* supported by a significant pattern of isolation by distance. On the other hand, this clustering or grouping of genotypes could enable the identification of diverse parents for breeding programs to create segregating offspring with high genetic diversity to conduct selection programs (Verma et al., 2019).

### 4.2 Conservation strategies for *K. odoratissima*

It is crucial to implement *in-situ* and ex-situ preservation of genetic diversity to guarantee the long-term persistence and evolution of populations, especially for endangered species (Liu et al., 2019; Zhong et al., 2019; Wu et al., 2020). Quantifying the genetic diversity and structure of native and threatened species is necessary for compiling scientific and rational conservation techniques, and further explicating mechanisms and causes of the rarity or extinction of these plants (Liu et al., 2019; Zhong et al., 2019; Wu et al., 2020). For instance, knowledge on the level of genetic diversity has fundamental role in establishing nature reserves to protect endangered species, in which can readily be applied for *K. odoratissima* (Fonseca et al., 2019). Providing economic and technical supports from the government, in order to hand over conservation matters to the local people, and instructing them about the ecology of plant and establishing controlled farms will be effective measures to reduce the overharvesting from the natural habitats. Several factors including narrow distribution range, restricted habitat preference, small population size, limited genetic diversity, slow growth rate, and reproductive system of the generative type are associated to the vulnerability of medicinal plants in response to exploitation pressure (Chen et al., 2016). In fact, *K. odoratissima* is a high-demand medicinal plant with narrow ecological niche, weak reproductive ability, and lack of broad wings for very effective long-distance seed dispersal (Mozaffarian, 2003; Ahmadi et al., 2021). Although regeneration of plants from root is the major determinates of population size of *K. odoratissima*, the recruitment of new individuals from successful seed germination is necessary for long term persistence of natural populations. Our observations revealed that habitats for *K. odoratissima* were confined to some fragmented, independent, and narrow ranges in alpine regions of Zagros. The plant has disappeared from numerous historical sampling locations as a result of excessive exploitation, grazing, and land cleanup (Ahmadi et al., 2021). Moreover, due to their seed dormancy, low germination rate, and inherently poor regeneration (Ahmadi et al., 2021), the population size and population density of *K. odoratissima* has significantly decreased in recent years (Jahantab et al., 2015). Thus, in order to mediate population decline, it is crucial to apply effective conservation measures.

*Kelussia odoratissima* is currently regarded as a state-class conserved species by the Iran Department of Natural Resources and Watershed Management Organization, and it is illegal to exploit natural resources of endangered species, but these measures are insufficient because the punishment imposed by the law is extremely negligible compared to the profit obtained from the smuggling the plant materials. The Natural Resources and Watershed Management Organization of Iran has also taken minor efforts to involve local people and conservationists as beneficiaries to encourage them to revive and preserve some habitats for the *K. odoratissima*. The potential for medicinal plants to adapt to their growing environments, are important factors in determining the effectiveness of *in-situ* conservation (Chen et al., 2016).

Regarding the preservation of medicinal plants, various sets of guidelines including offering both *in-situ* and ex-situ conservation have been developed. The establishment of natural reserves and wild nurseries are common examples of preserving a medicinal properties of plants in their natural environment, while botanical gardens and seed banks serve as significant models for ex-situ conservation and future reintroduction (Chen et al., 2016).

The capability to conserve more diversity is more significant through *in-situ* conservation which is mainly facilitated by protected areas and applying an ecosystem-oriented approach together with its extensive network of relationships. Natural reserves are regions of significant wild resources that are protected and were established to preserve and restore biodiversity (Chen et al., 2016). According to some experts, this strategy is a ready-made method to preserve wild relatives of crop plants in their natural habitats (Bönisch et al., 2020). Thus, establishing natural reserves will also protect dominant vegetative types of the *K. odoratissima* habitats are *Astragalus adscendens* Boiss. & Hausskn. And *Fritillaria imperialis* L. (Ghasemi Nafchi, 2015). A wild nursery with a species-oriented approach is brought about to cultivate and domesticate threatened medicinal plants in a protected region, natural habitat, or a location close to where the plants grow naturally (Chen et al., 2016). Due to the fact that most genetic variation frequently occurs within populations, it is crucial to prioritize *in-situ* conservation for populations with significant genetic diversity and those with unique genes (Aboukhalid et al., 2017; Wu et al., 2020; Chen et al., 2021). The high genetic diversity and low inbreeding coefficient of these populations suggest that they may still have some remnants of their archaic genetic structure. Aboukhalid et al. (2017) expressed that the high level of genetic diversity might suggest that habitat fragmentation and overexploitation have not yet severely affected the diversity within the populations. In contrast, populations with lower levels of genetic diversity are likely to have lower chance of survival in their natural habitats in the future, probably due to their relatively high levels of inbreeding depression.

In addition to *in-situ* conservation, the practice of ex-situ conservation plays a valuable role in conserving medicinal plant resources, particularly for a rare threatened species experiencing overharvesting, slow growth and susceptibility to diseases (Chen et al., 2016). Botanical gardens with help of seed banks can have a further role in the ex-situ conservation of medicinal plants by implementing strategies for domestication and breeding, and also developing propagation and cultivation procedures, with the aim of supplying sufficient materials for germplasm innovation and the potential future reintroduction of these native species (Chen et al., 2016; Aboukhalid et al., 2017; Chen et al., 2021). Populations with small sizes or limited numbers of reproducing individuals will definitely encounter an abrupt fall in genetic diversity and elevated extinction risk (Wu et al., 2020). As a result, it is urgently necessary to collect plant samples to establish a germplasm resource library. 

Majority of the medicinal plants are indigenous species, and their therapeutic characteristics are associated to the presence of secondary metabolites that are produced in response to environmental stimuli in their natural habitats and may not necessarily expressed under cultivation conditions (Chen et al., 2016). However, it is better to reduce the demand for these valuable medicinal plants through domestication and cultivation, such approaches have not been applied concerning *K. odoratissima* so far. Cultivation under controlled growth environment can enhance the yield of secondary metabolites and sustainable production, thereby helping to reduce the quantity of harvested medicinal plants from their natural range (Chen et al., 2016; Aboukhalid et al., 2017).

## 5 Conclusion

The results obtained from this study has provided fundamental data on the genetic diversity and structure of natural populations of *K. odoratissima* for the first time. This knowledge might be valuable for formulating long-term plans to protect and to manage this rare medicinal plant. The results demonstrated that the microsatellite markers comparatively have great efficacy in differentiating different populations of *K. odoratissima*. Despite the species narrow distribution range, natural populations still exhibit significant levels of genetic diversity. This information will also provide opportunities in the direction of implementation of concrete plans for *in-situ* and ex-situ conservation. In addition, the results might help in choosing populations from different genetic clusters to screen desirable traits in terms of usefulness in pharmacologically commercial applications, and thus shed light on the process of breeding and domestication of *K. odoratissima*. On the other hand, these novel markers can be used for conducting population genetics studies on other threatened species closely related to *Kelussia* through cross-amplification of introduced loci.

## Data availability statement

The sequence data of developed microsatellite loci presented in the study are deposited in the GeneBank, accession numbers OQ992688- OQ992710.

## Author contributions

FM: Data curation, Formal analysis, Investigation, Methodology, Software, Visualization, Writing – original draft. M-TE: Investigation, Conceptualization, Funding acquisition, Project administration, Supervision, Writing – review & editing. AS: Resources, Writing – review & editing, Investigation, Methodology. MA: Writing – review & editing. MF-A: Conceptualization, Formal Analysis, Investigation, Methodology, Project administration, Software, Supervision, Validation, Visualization, Writing – review & editing.
