# Supplementary material for: Genetic variation and structure of endemic and endangered wild celery (Kelussia odoratissima Mozaff.) quantified using novel microsatellite markers developed by next-generation sequencing
Source: Front Plant Sci. 2024 Apr 4;15:1301936. doi: 10.3389/fpls.2024.1301936 (PMC11024376; doi:10.3389/fpls.2024.1301936)
Supplement: Supplementary file 1 [file Presentation_1.pdf]

Supplementary material

Genetic Variation and Structure of Endemic and Endangered Wild Celery (*Kelussia odoratissima* Mozaff.) Quantified Using Novel Microsatellite Markers Developed by Next-Generation Sequencing

F. Mahdavikia [1] , M.T. Ebadi[1] , A. Shojaeiyan[1] , M. Ayyari[1] and M. Falahati-Anbaran[2]

[1]Department of Horticultural Science, Faculty of Agriculture, Tarbiat Modares University (TMU), Tehran, Iran

[2]NTNU University Museum, Norwegian University of Science and Technology, Trondheim, Norway

Corresponding authors:

mt.ebadi@modares.ac.ir

falahati@ntnu.no

Table S1. Characteristics for microsatellite loci tested for amplification and introduced for *K. odoratissima*. Dash line (-) indicates no amplification was found for the locus

Locus name	Primer sequences (5'-3')	Motif	Tm (°C)	Number of alleles (Na)	Expected amplicon size (bp)	Polymorphic Information Content (PIC)	GenBank accession No.
K.odora2	F: ACGAAACCAAGAACACAGCC<br>R: CCATGTGGTGTCAAGGAGTG	(AC)8	53	-	97	-	-
K.odora3	F: TTCGAGTCTCGCCTTCCTAA<br>R: CCAAAATAGGCCTTCCGTTT	(AC)16	64	12	137	0.854	OQ992688
K.odora4	F: GGGTGGTTTGGTTCAAAATG<br>R: CACTTCCACGGTACCATCACT	(AC)10	64	9	219	0.847	OQ992689
K.odora5	F: ATGCTCGGTCCACTATCCAG<br>R: CTGAGCCACAGCAGACAGAG	(CA)5	64	9	111	0.8	OQ992690
K.odora6	F: CCAGAATTTTGGTGCCAGTT<br>R: TACACCTGCCTGCTTTTGTT	(CA)11	64	7	191	0.669	OQ992691
K.odora7	F: TTCGGGGCATTGTCTCTATC<br>R: AGCCCAGGTGCTGATTTATG	(CA)7	64	10	308	0.798	OQ992692
K.odora8	F: TTGCTGACAACAGACACAGTC<br>R: TGTTGAAACTCCACAGAAACCA	(TG)6	64	8	102	0.766	OQ992693
K.odora9	F: CAACTTGGCTGAAGGAAGAAA<br>R: TCAGATAAAATTCTCTTTTGCTGC	(TG)7	62	11	148	0.797	OQ992694
K.odora10	F: GGAATATCAACTTTGTTTTCCCA<br>R: ACACATTCCAGTCTGGGGAG	(GT)9	64	8	171	0.772	OQ992695
K.odora11	F: CACCGTGTATTCTACCTTCTTCAC<br>R: AACCCAAATGAAGAAAGCCTG	(TC)11	64	7	90	0.675	OQ992696
K.odora12	F: AGGTTGCCCCTTGTTTTACA<br>R: GCATCATCCTCTCAAAAGGG	(TC)11	63	11	218	0.835	OQ992697
K.odora13	F: TTCATTTCGGAACCTTCTTATC<br>R: GGTTTAAACGGTAGGGAGGC	(CT)9	63	9	113	0.774	OQ992698
K.odora14	F: TGATTCTTGACTTGGCATTTG<br>R: GGACAAAATGTGGCAAGGAT	(CT)10	62	10	168	0.834	OQ992699
K.odora15	F: TTCGACGGGTTTTATCAAGG<br>R: CTCTCTCCTCCGTCTCTGTTCT	(AG)9	63	14	232	0.876	OQ992700
K.odora16	F: TCGTTTGAGTATGGAAAAGTGAAA<br>R: CCCGATCTATAGACACAATCTAACA	(GA)11	63	7	102	0.793	OQ992701
K.odora17	F: CGTTCCAACAAAAATATGACCA<br>R: CTCTTGCTGGACAATCCCTC	(TA)5	53	-	129	-	-
K.odora18	F: TTGAATGTGCGGACTTAATCA<br>R: TGTGTCTAACTCTTTTCTACGTTTCA	(TTC)6	63	11	145	0.831	OQ992702

K.odora19	F: CGATATCATCATAACCATCATCTTC<br>R: TGCAACAAGAAGTCGTCCAC	(TTC)7	64	8	251	0.8	OQ992703
K.odora20	F: TGGTTGTTTTTCGTTCTGTCTG<br>R: TGGTCTATGGCACGATACCC	(AAG)6	63	8	96	0.829	OQ992704
K.odora21	F: TTTGAACTTCGGCGTCTCTT<br>R: TCATGTAATCAGAACCCGGA	(AAG)5	64	27	179	0.935	OQ992705
K.odora22	F: CCTTCAGCAACAAACTTCCC<br>R: GATCCCTTGAACTCTGGTGG	(AAC)10	64	12	150	0.846	OQ992706
K.odora23	F: CCAGAACCATGCCTCCTAAA<br>R: CTCCATTTCTTTCAGCCAGG	(CAA)6	56	15	262	0.874	OQ992707
K.odora24	F: CATCGGTATCGTCATCTTCCT<br>R:TCGAATTGTAAGAGATCAAAATAGTTG	(TCT)10	64	10	122	0.792	OQ992708
K.odora25	F: CATTTCTAAAGCTTCAAATGATCTAC<br>R: GCTCCTCTAGCCTGAGCAAA	(AGG)5	64	7	193	0.801	OQ992709
K.odora26	F: TCAAACCATTGACGAAAGCA<br>R:GATATTACACAGGAAATGTAGAACTCA	(GAT)5	63	20	317	0.918	OQ992710

Dash line (-) indicates no amplification was found for the locus.

Table S2. The frequency and distribution of di-, tri and tetra nucleotide repeats

Motif	Frequency of repetitive motif								Total	%
	**5**	**6**	**7**	**8**	**9**	**10**	**11**	**>11**		
Di-	45	17	23	13	15	6	6	8	133	68.21
Tri-	31	16	6	3	1	3	0	0	60	30.77
Tetra-	1	0	0	0	0	0	0	1	2	1.03
Total	77	33	29	16	16	9	6	9	195	100
%	39.49	16.92	14.87	8.21	8.21	4.62	3.08	4.62	100	

Table S3. Within-population genetic statistics of 23 microsatellite loci in *K. odoratissima*. Na, H_O, H_E, r, P, N_E, R_S, R_P, F_{IS}, and * denote the number of alleles, observed and expected heterozygosities, frequency of null alleles, *P*-value for the HW equilibrium test, effective number of alleles, private allelic richness, inbreeding coefficient, respectively.

Population	Parameter	K.odora03	K.odora04	K.odora05	K.odora06	K.odora07	K.odora08	K.odora09	K.odora10	K.odora11	K.odora12	K.odora13	K.odora14	
**Sras-I**	Na	4.000	4.000	5.000	3.000	4.000	3.000	4.000	6.000	4.000	6.000	3.000	4.000	
	H_O	1.000	1	0.5	0.13	1	0.38	0.88	1	1	1	0.75	0.86	
	H_E	0.77	0.63	0.84	0.57	0.75	0.7	0.71	0.82	0.65	0.86	0.63	0.77	
	P	0.0021	0.0025	0.0001	0.007	0.0571	0.007	0.9105	0.0158	0.1173	0.6793	0.0216	0.7016	
	r	0.000	0.000	0.1476	0.2467	0.000	0.1556	0.000	0.000	0.3553	0.000	0.0298	0.000	
	N_E													3.563
	R_S													3.7
	R_P													0.08
	F_{IS}													-0.105
**Sras-II**	Na	6.000	4.000	7.000	4.000	4.000	6.000	4.000	5.000	3.000	6.000	4.000	4.000	
	H_O	1.000	1	1	0.38	0.33	0.63	0.88	1	1	1	0.88	0.88	
	H_E	0.880	0.79	0.86	0.71	0.47	0.86	0.77	0.72	0.63	0.82	0.63	0.71	
	P	0	0.0015	0.9439	0.0188	0.2727	0.0096	0.0712	0.242	0.0626	0.0174	0.0216	0.0335	
	r	0.000	0.000	0.000	0.1556	0.000	0.1181	0.000	0.000	0.3232	0.000	0.000	0.000	
	N_E													3.876
	R_S													4.05
	R_P													0.14
	F_{IS}													-0.099
**Sras-III**	Na	5.000	4.000	5.000	3.000	2.000	4.000	5.000	3.000	4.000	6.000	3.000	6.000	
	H_O	0.875	1	1	0	0.33	0.88	1	1	1	1	0.75	1	
	H_E	0.84	0.63	0.79	0.75	0.3	0.77	0.79	0.62	0.68	0.8	0.63	0.87	
	P	0.0825	0.0025	0.1806	0.0003	1	0.0019	0.0703	0.0909	0.3038	0.0001	0.0216	0.071	
	r	0.000	0.000	0.000	0.5643	0.000	0.000	0.000	0.000	0.3869	0.000	0.0298	0.000	
	N_E													3.497

	R_S															3.67
	R_P															0.23
	F_{IS}															-0.126
**Brgn**	Na	4.000	6.000	4.000	4.000		3.000		2.000	3.000	5.000	3.000	5.000	3.000	4.000	
	H_O	0.75	1	1	0.38		0.38		1	1	1	1	1	0	0.88	
	H_E	0.75	0.83	0.72	0.77		0.52		0.5	0.63	0.82	0.63	0.8	0.61	0.71	
	P	0.0027	0.0048	0.5823	0.0033		0.1282		0.0253	0.0626	0.0074	0.0626	0.0012	0.0014	0.2727	
	r	0.000	0.000	0.000	0.2106		0.1132		0.000	0.000	0.000	0.3232	0.000	0.3553	0.000	
	N_E															3.382
	R_S															3.57
	R_P															0.13
	F_{IS}															-0.147
**Abdz**	Na	5.000	2.000	5.000	2.000		4.000		4.000	6.000	5.000	5.000	6.000	5.000	4.000	
	H_O	0.875	1	0.88	0		0.67		0.63	1	1	1	1	0.75	1	
	H_E	0.8	0.5	0.8	0.54		0.8		0.8	0.84	0.73	0.78	0.86	0.66	0.79	
	P	0	0.0253	0.0132	0.007		0.1688		0.0031	0.0115	0.0242	0.2307	0.0026	0.3786	0.0015	
	r	0.125	0.000	0.000	0.3232		0.000		0.3869	0.000	0.000	0.2627	0.000	0.000	0.000	
	N_E															3.712
	R_S															3.87
	R_P															0.14
	F_{IS}															-0.147
**Klse**	Na	2.000	2.000	5.000	2.000		3.000		3.000	6.000	4.000	3.000	4.000	2.000	7.000	
	H_O	1	1	1	0		0.6		1	1	1	1	1	0.38	1	
	H_E	0.5	0.5	0.76	0.54		0.7		0.56	0.79	0.71	0.63	0.71	0.32	0.88	
	P	0.0253	0.0253	0.0236	0.007		0.1111		0.0253	0.0428	0.0949	0.0626	0.0041	1	0.152	
	r	0.000	0.000	0.000	0.3232		0.0719		0.000	0.000	0.000	0.3232	0.000	0.000	0.000	
	N_E															3.361
	R_S															3.51
	R_P															0.15

	F_{IS}													-0.264
**Drsp**	Na	5.000	2.000	5.000	3.000	7.000	4.000	6.000	3.000	4.000	6.000	5.000	7.000	
	H_O	0.75	1	0.86	0	0.5	0.38	1	1	0.88	1	0.5	0.88	
	H_E	0.84	0.5	0.8	0.68	0.88	0.61	0.8	0.56	0.73	0.84	0.75	0.86	
	P	0.0034	0.0253	0.9126	0.0008	0.0137	0.007	0.0213	0.0253	0.0151	0.0103	0.0625	0.2709	
	r	0.000	0.000	0.000	0.3748	0.2221	0.000	0.000	0.000	0.3748	0.000	0.0895	0.0659	
	N_E													3.546
	R_S													3.7
	R_P													0.1
	F_{IS}													-0.125
**Vstg**	Na	4.000	4.000	6.000	4.000	2.000	5.000	5.000	4.000	4.000	7.000	3.000	4.000	
	H_O	1	1	1	0.13	0.8	0.75	1	1	1	1	0.88	0.86	
	H_E	0.63	0.79	0.85	0.61	0.5	0.77	0.71	0.75	0.69	0.87	0.55	0.79	
	P	0.0025	0.0015	0.2157	0.0014	0.4286	0.0514	0.3092	0.0751	0.2541	0.0025	0.1385	0.0154	
	r	0.000	0.000	0.000	0.2646	0.000	0.000	0.000	0.000	0.3966	0.000	0.000	0.000	
	N_E													3.555
	R_S													3.75
	R_P													0.1
	F_{IS}													-0.193
**Khgn**	Na	7.000	4.000	5.000	3.000	2.000	4.000	4.000	2.000	4.000	4.000	2.000	5.000	
	H_O	1	1	1	0	0.29	0.88	1	1	1	1	0.75	0.83	
	H_E	0.88	0.74	0.81	0.61	0.26	0.71	0.76	0.5	0.71	0.79	0.48	0.82	
	P	0.1827	0.0043	0.012	0.0014	1	0.0141	0.1784	0.0253	0.0949	0.0015	0.4406	0.0822	
	r	0.000	0.000	0.000	0.3553	0.000	0.000	0.000	0.000	0.2696	0.000	0.000	0.000	
	N_E													3.681
	R_S													3.83
	R_P													0.12
	F_{IS}													-0.24
**Durk**	Na	4.000	2.000	4.000	4.000	0.000	2.000	5.000	5.000	6.000	6.000	4.000	4.000	

	H_O	0.875	1	0.38	0.13	-	0.38	1	1	1	1	0.57	1	
	H_E	0.77	0.5	0.63	0.73	-	0.32	0.76	0.73	0.84	0.84	0.71	0.77	
	P	0.0059	0.0253	0.0713	0.0008	-	1	0.0085	0.159	0.0441	0.0005	0.1096	0.0004	
	r	0.000	0.000	0.0926	0.3748	No information (No full genotype)	0.000	0.000	0.000	0.2043	0.000	0.0476	0.000	
	N_E													3.240
	R_S													4.33
	R_P													0.1
	F_{IS}													-0.118
**Dlfz**	Na	4.000	6.000	3.000	5.000	3.000	3.000	5.000	4.000	5.000	4.000	6.000	5.000	
	H_O	1	1	1	0.5	0.2	1	1	1	1	1	0.63	1	
	H_E	0.77	0.73	0.63	0.86	0.55	0.61	0.8	0.71	0.71	0.77	0.82	0.76	
	P	0.0004	0.0014	0.0626	0.0081	0.1111	0.0601	0.3678	0.0041	0.0202	0.0234	0.0175	0.0087	
	r	0.000	0.000	0.000	0.155	0.1983	0.000	0.000	0.000	0.2646	0.000	0.0345	0.000	
	N_E													3.798
	R_S													3.91
	R_P													0.18
	F_{IS}													-0.181
**Ghrn**	Na	6.000	4.000	5.000	4.000	1.000	2.000	4.000	3.000	6.000	8.000	5.000	3.000	
	H_O	1	1	0.88	0.13	0	0	1	1	1	1	0.63	1	
	H_E	0.77	0.63	0.84	0.73	0	0.54	0.74	0.56	0.84	0.89	0.77	0.63	
	P	0.1724	0.0025	0.0001	0.0005	-	0.007	0.0157	0.0253	0.048	0.0908	0.1098	0.0626	
	r	0.000	0.000	0.000	0.3155	No information (Less than 2 alleles)	0.3232	0.000	0.000	0.1858	0.000	0.0719	0.000	
	N_E													3.607
	R_S													3.74
	R_P													0.56
	F_{IS}													-0.119

Table S3. Continued

Population	Parameter	K.odora15	K.odora16	K.odora18	K.odora19	K.odora20	K.odora21	K.odora22	K.odora23	K.odora24	K.odora25	K.odora26	
**Sras-I**	Na	3.000	2.000	5.000	3.000	3.000	7.000	4.000	6.000	5.000	7.000	5.000	
	H_O	1	1	1	1	0	1	1	0.5	0.88	1	0.13	
	H_E	0.64	0.5	0.8	0.64	0.73	0.87	0.79	0.8	0.8	0.87	0.84	
	P	0.0552	0.0909	0.0014	0.0552	0.0043	0.1992	0.0015	0.0109	0.0702	0.0637	0	
	r	0.000	0.000	0.000	0.000	0.3816	0.000	0.000	0.1058	0.000	0.000	0.354	
	N_E												3.563
	R_S												3.7
	R_P												0.08
	F_{IS}												-0.079
**Sras-II**	Na	7.000	5.000	6.000	4.000	3.000	9.000	7.000	6.000	3.000	6.000	6.000	
	H_O	1	1	1	0.88	0	1	0.5	1	1	1	0.29	
	H_E	0.8	0.79	0.84	0.73	0.76	0.91	0.85	0.84	0.57	0.79	0.71	
	P	0.0578	0.002	0.0423	0.1422	0.001	0.743	0.0104	0	0.049	0.0331	0.001	
	r	0.000	0.000	0.000	0.000	0.3957	0.000	0.125	0.000	0.000	0.000	0.1921	
	N_E												3.876
	R_S												4.05
	R_P												0.14
	F_{IS}												-0.073
**Sras-III**	Na	7.000	4.000	6.000	3.000	2.000	6.000	3.000	7.000	4.000	4.000	4.000	
	H_O	1	1	1	0.88	0	1	0.13	0.88	0.88	1	0.25	
	H_E	0.89	0.79	0.75	0.59	0.33	0.83	0.52	0.87	0.73	0.71	0.8	
	P	0.0357	0.0015	0.1113	0.3287	0.0909	0.0076	0.0154	0.0036	0.0151	0.0041	0.0012	
	r	0.000	0.000	0.000	0.000	0.2512	0.000	0.2427	0.000	0.0833	0.000	0.3869	
	N_E												3.497
	R_S												3.67
	R_P												0.23
	F_{IS}												-0.073
**Brgn**	Na	7.000	5.000	4.000	3.000	2.000	6.000	3.000	6.000	4.000	4.000	5.000	
	H_O	1	1	1	1	0	1	0.33	0.63	1	1	0.88	
	H_E	0.88	0.81	0.77	0.56	0.43	0.77	0.57	0.89	0.79	0.71	0.77	
	P	0.2019	0.0022	0.0004	0.0253	0.0154	0.0778	0.0303	0	0.0021	0.0041	0.0364	
	r	0.000	0.000	0.000	0.000	0.2903	0.000	0.315	0.1181	0.000	0.000	0.1884	
	N_E												3.382
	R_S												3.57
	R_P												0.13

	F_{IS}												-0.108
**Abdz**	Na	6.000	5.000	5.000	2.000	2.000	8.000	4.000	5.000	4.000	6.000	7.000	
	H_O	0.88	1	1	0.83	0	1	0.17	0.75	1	1	0.67	
	H_E	0.83	0.82	0.78	0.5	0.33	0.88	0.73	0.79	0.79	0.83	0.85	
	P	0.0815	0.0078	0.0722	0.3939	0.0909	0.8764	0.0043	0	0.0021	0.0823	0.1506	
	r	0.000	0.000	0.000	0.000	0.2512	0.000	0.2724	0.000	0.000	0.000	0.000	
	N_E												3.712
	R_S												3.87
	R_P												0.14
	F_{IS}												-0.044
**Klse**	Na	5.000	4.000	5.000	4.000	5.000	5.000	5.000	6.000	3.000	3.000	5.000	
	H_O	1	1	0.88	1	0.63	1	1	1	0.75	1	0.38	
	H_E	0.83	0.79	0.82	0.71	0.82	0.71	0.82	0.85	0.63	0.64	0.84	
	P	0.0754	0.0021	0.0432	0.0996	0.0005	0.3094	0.0052	0.0507	0.0216	0.0552	0.0007	
	r	0.000	0.000	0.000	0.000	0.1451	0.000	0.000	0.000	0.0298	0.000	0.2195	
	N_E												3.361
	R_S												3.51
	R_P												0.15
	F_{IS}												-0.244
**Drsp**	Na	5.000	6.000	4.000	4.000	2.000	6.000	4.000	7.000	2.000	3.000	4.000	
	H_O	1	1	1	0.75	1	1	0.63	0.88	0.86	0.71	0.88	
	H_E	0.77	0.84	0.63	0.75	0.5	0.8	0.77	0.89	0.5	0.64	0.73	
	P	0.1016	0.0001	0.0025	0.3606	0.0253	0.3061	0.029	0.0402	0.1608	0.0303	0.0151	
	r	0.000	0.000	0.000	0.000	0.000	0.000	0.0158	0.000	0.000	0.0384	0.000	
	N_E												3.546
	R_S												3.7
	R_P												0.1
	F_{IS}												-0.147
**Vstg**	Na	5.000	7.000	5.000	4.000	3.000	9.000	2.000	8.000	4.000	4.000	5.000	
	H_O	0.86	1	1	0.88	0.38	1	0.13	1	0.67	1	0.38	
	H_E	0.82	0.78	0.76	0.71	0.68	0.92	0.13	0.89	0.63	0.74	0.75	
	P	0.0116	0.0046	0.0077	0.2727	0.0054	0.2255	-	0	0.2121	0.0043	0.0113	
	r	0.000	0.000	0.000	0.000	0.3333	0.000	0.000	0.000	0.000	0.000	0.1982	
	N_E												3.555
	R_S												3.75
	R_P												0.1
	F_{IS}												-0.148
**Khgn**	Na	7.000	4.000	6.000	4.000	2.000	9.000	7.000	8.000	4.000	4.000	8.000	

	H_O	1	1	1	1	0	1	1	1	1	1	0.75	
	H_E	0.85	0.77	0.79	0.69	0.43	0.88	0.86	0.88	0.64	0.71	0.89	
	P	0.1691	0.0004	0.0398	0.2541	0.0154	0.0423	0.0668	0.2551	0.007	0.0041	0.0037	
	r	0.000	0.000	0.000	0.000	0.2903	0.000	0.000	0.000	0.000	0.000	0.000	
	N_E												3.681
	R_S												3.83
	R_P												0.12
	F_{IS}												-0.177
**Durk**	Na	7.000	4.000	5.000	3.000	2.000	7.000	2.000	8.000	4.000	5.000	4.000	
	H_O	0.88	1	1	1	0	1	0.25	1	0.75	1	0.63	
	H_E	0.86	0.63	0.79	0.63	0.57	0.84	0.23	0.88	0.77	0.8	0.66	
	P	0.0198	0.0025	0.0195	0.0626	0.0054	0.1123	1	0.0669	0.025	0.001	0.049	
	r	0.000	0.000	0.000	0.000	0.3333	0.000	0.000	0.000	0.000	0.000	0.000	
	N_E												3.240
	R_S												3.61
	R_P												0.1
	F_{IS}												-0.104
**Dlfz**	Na	8.000	4.000	4.000	3.000	3.000	10.000	5.000	8.000	5.000	4.000	4.000	
	H_O	1	1	1	1	0	1	0.5	1	1	1	0.29	
	H_E	0.93	0.63	0.71	0.61	0.52	0.93	0.8	0.91	0.76	0.77	0.73	
	P	0.0301	0.0025	0.1528	0.0601	0.007	1	0.0087	0.0741	0.0084	0.0004	0.0256	
	r	0.000	0.000	0.000	0.000	0.3276	0.000	0.1393	0.000	0.000	0.000	0.2209	
	N_E												3.798
	R_S												3.91
	R_P												0.18
	F_{IS}												-0.122
**Ghrn**	Na	8.000	5.000	5.000	3.000	3.000	7.000	3.000	8.000	5.000	4.000	6.000	
	H_O	1	1	1	1	0	1	1	1	1	1	0.5	
	H_E	0.91	0.76	0.73	0.61	0.68	0.85	0.63	0.89	0.68	0.71	0.8	
	P	0.2746	0.0053	0.0252	0.0601	0.0008	0.1196	0.0626	0.3367	0.01	0.0041	0.0572	
	r	0.000	0.000	0.000	0.000	0.3748	0.000	0.000	0.000	0.000	0.000	0.1058	
	N_E												3.607
	R_S												3.74
	R_P												0.56
	F_{IS}												-0.125

Table S4. Analysis of molecular variance (AMOVA) for natural populations of *K. odoratissima*

**Source of variance**	**Degree of freedom (df)**	**Sum of squares (SS)**	**Variance components**	**Percentage of variation**
Among Populations	11	238.984	0.93274 Va	12.06
Within Populations	180	1224.375	6.80208 Vb	87.94
Total	191	1463.359	7.73482	

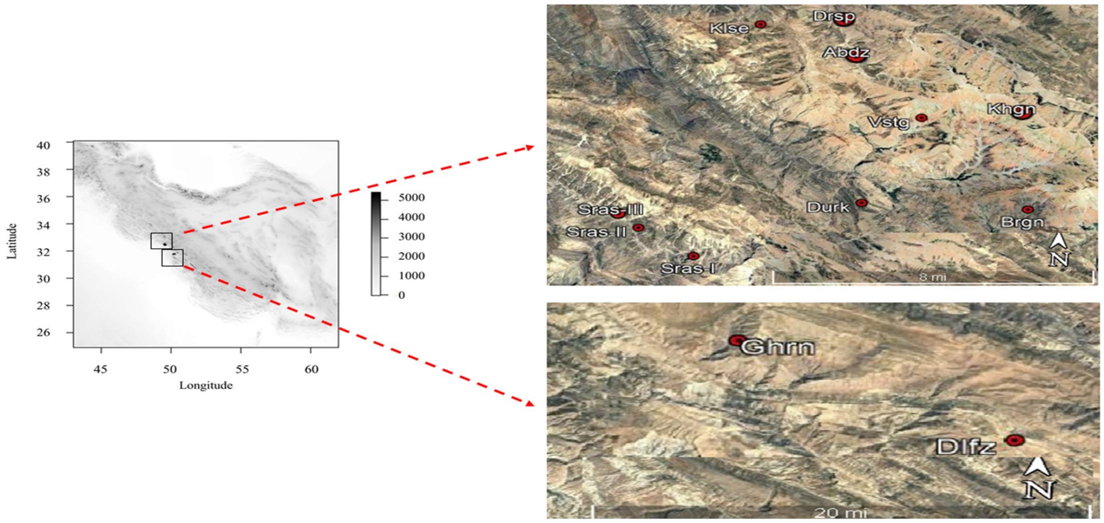

Fig. S1. The sampling sites of the natural populations of *Kelussia odoratissima* Mozaff.. Localities of sampling SarAghaSeyed-I (Sras-I), SarAghaSeyed-II (Sras-II), SarAghaSeyed-III (Sras-III), Birahgan (Brgn), Abdoz (Abdz), Kelose (Klse), DareSepestan (Drsp), Vestegan (Vstg), Kahgan (Khgn), Durak (Durk), DelAfruz (Dlfz), and Gharun (Ghrn) are marked with circles.

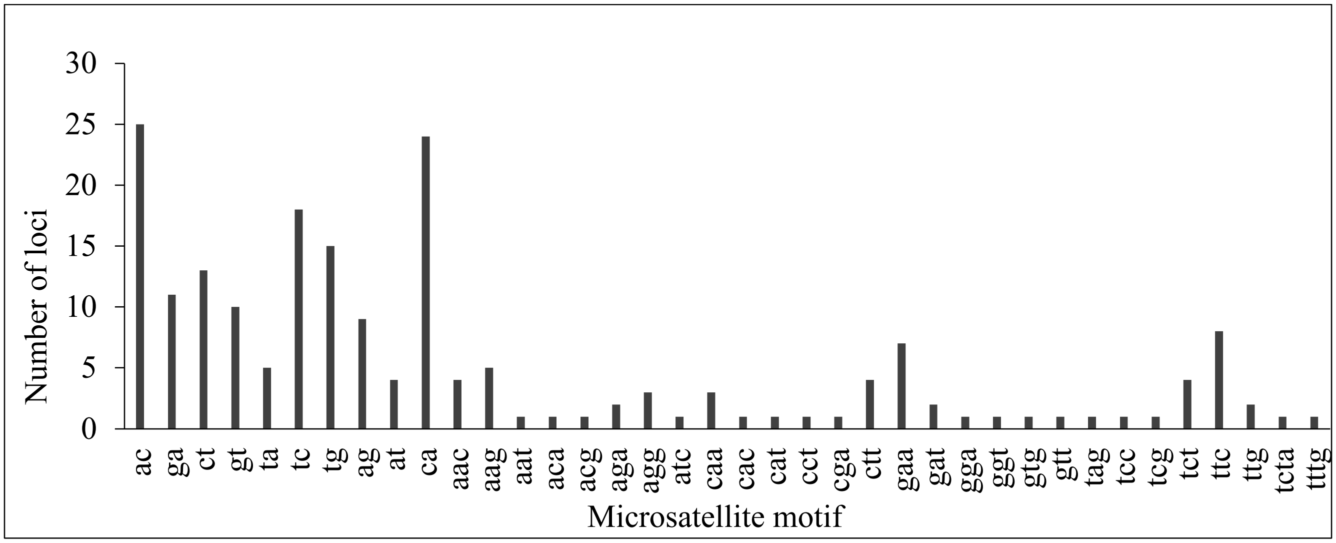

Fig S2. The nucleotide composition and the frequency of microsatellite motifs across the best bioinformatically confirmed loci.

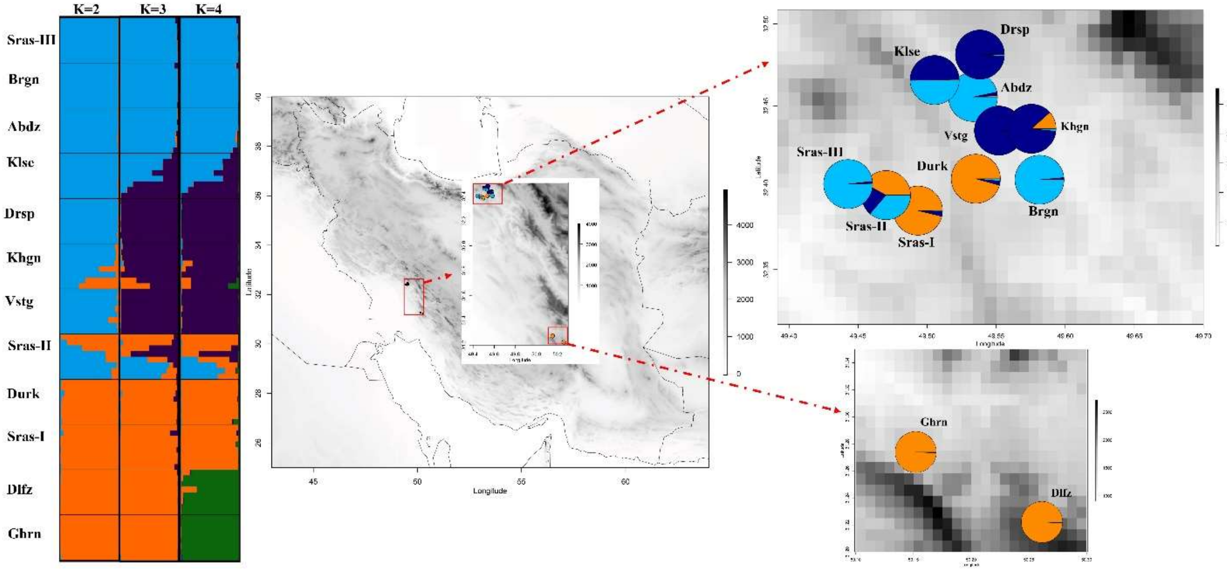

Fig. S3. The genetic structure of 96 individuals from natural populations of *Kelussia odoratissima* Mozaff.. The membership proportion of individuals (left) and populations (right) to each genetic cluster. The matrix of membership to each genetic cluster are obtained from summarizing the outputs of 16 separate runs with high similarity scores (K2= 0.998, and K3=0.995).

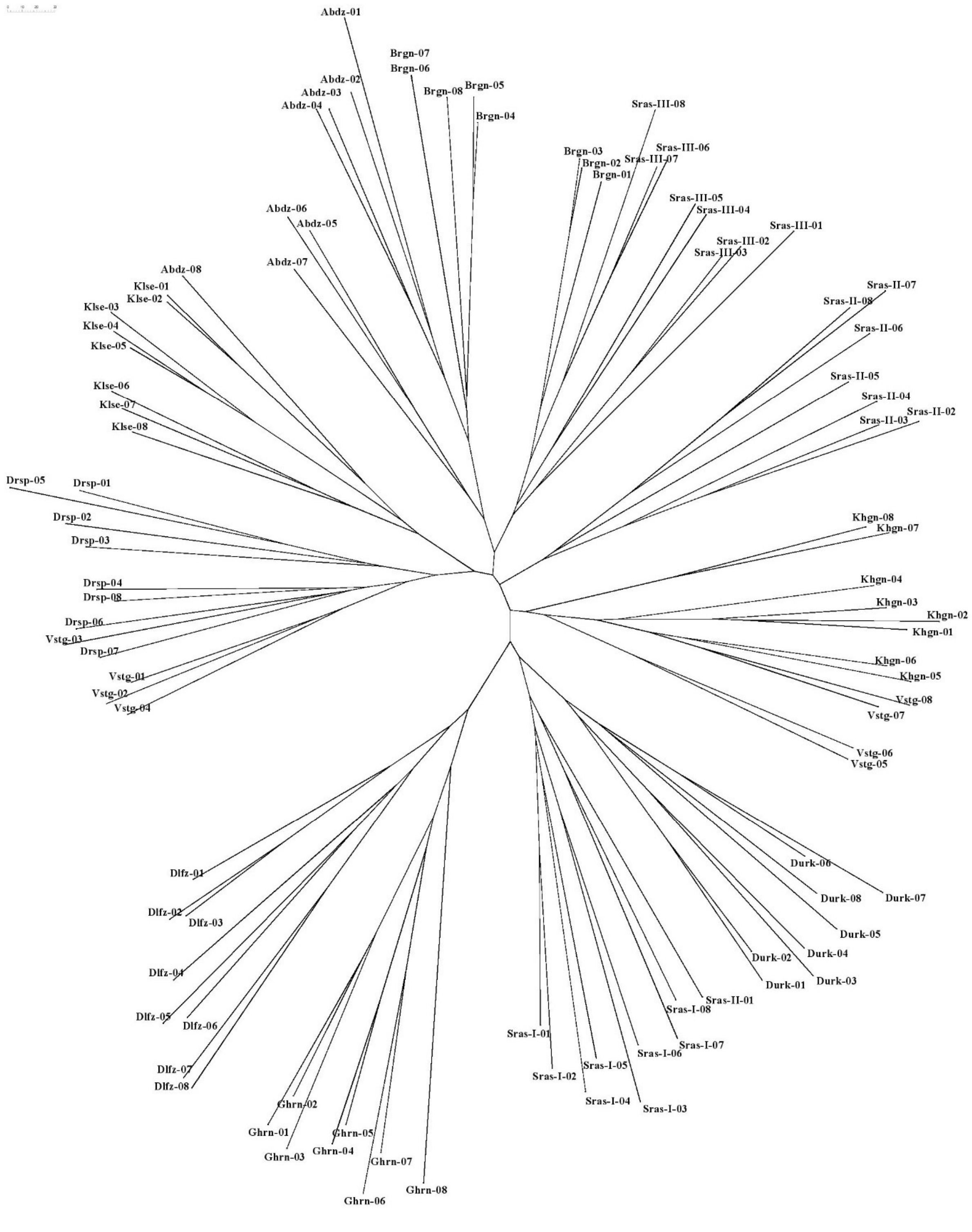

Fig. S4. Neighbor-net network representing the genetic relationships between 96 individuals from 12 wild populations of *Kelussia odoratissima*
